# HRQoL assessment by SRS-30 for Chinese patients with surgery for Adolescent Idiopathic Scoliosis (AIS)

**DOI:** 10.1186/1748-7161-10-S2-S19

**Published:** 2015-02-11

**Authors:** Bobby Kin Wah Ng, Wai-Wang Chau, Chak-Na Hui, Po-Yin Cheng, Chau-Yuet Wong, Bin Wang, Jack Chun Yiu Cheng, Tsz Ping Lam

**Affiliations:** 1 Department of Orthopaedics and Traumatology, Chinese University of Hong Kong, Hong Kong, China; 2 Joint Scoliosis Research Center of the Chinese University of Hong Kong and Nanjing University, Nanjing, China; 3 Paediatric Surgical and Orthopaedic Wards, Prince of Wales Hospital, Hong Kong, China

**Keywords:** Quality of Life, Questionnaires, Adolescent, Scoliosis, Asian Continental Ancestry Group

## Abstract

**Introduction:**

Health-related quality of life (HRQoL) outcome questionnaire, Scoliosis Research Society (SRS)-30, had been well received since its establishment in 2003. Literatures from Asia on the use of SRS-30 mainly focused on the translation process and validation process, but not on measuring outcomes, particularly in the Chinese community. We carried out a prospective cohort study to evaluate the HRQoL of Chinese AIS adolescents with severe scoliosis after surgery.

**Methods:**

One hundred and four Chinese AIS patients with severe scoliosis undergoing posterior spinal fusion between 2009 and 2013 were recruited in this study. They completed SRS-30 questions before surgery, before hospital discharge, and at follow-up. Mean scores and percentages of individual scores in different domains, and composite scores in terms of subtotal and total scores were calculated referring to the scoring system. Gender-specific and period-specific descriptive analyses were described. Correlation of mean domain scores at the three time points were explored to look for any time-specific relationship. Linear regression analysis looking for potential risk factors on domain scores at different time points by gender were also carried out.

**Results:**

Mean age was 16.28 at surgery, and 83.6% were female. Significant correlations between pre-op scores and scores after surgery were observed in function/activity domain (p=0.05) in males, and pain (p=0.04) and satisfaction with management (p=0.04) domains in females. No gender difference in all 5 domain scores at the 3 time points was found. Pre-op maximum Cobb angle and corrected angle were found to be risk factors on self-image, as well as satisfaction with management, in male and female patients.

**Conclusions:**

This is the first report on the evaluation of the clinical HRQoL outcomes of Chinese AIS patients with severe scoliosis after surgery. Medical professionals should pay attention to take care of the difference in personal perceptions of feelings between boys and girls. Special care should also be allocated to AIS patients, and try to arrange earlier surgical intervention.

## Introduction

Health-related quality of life (HRQoL) outcome questionnaire, Scoliosis Research Society (SRS)-30, has been well received since its establishment in 2003. Literatures from Asia on the use of SRS-30 mainly focused on the translation process and validation process, but not on measuring outcomes, particularly in the Chinese community.[1]

The Scoliosis Research Society-30 (SRS-30) questionnaire is well-known as a standard assessment tool to evaluate patients’ quality of life across five domains: function/activity, pain, self-image/appearance, mental health, and satisfaction with management.[2-4] Over the past decade, SRS-30 had been widely applied in different areas of quality of life of patients with different spinal deformities e.g. surgical waiting time[5], ethnic and cultural difference[6,7], satisfaction level on patients with Parkinson disease after surgery[8].

We carried out a prospective cohort study using SRS-30 to evaluate HRQoL for Chinese AIS adolescents before and after surgery.

## Materials and methods

One hundred and four (104) Chinese AIS patients (20 males and 84 females) undergoing posterior spinal fusion between 2009 and 2013 were recruited. They completed the SRS-30 questionnaire at 3 time points, 1) just before spinal surgery after hospital admission (pre-op), 2) before hospital discharge (discharge), and 3) 6 months to 3 years after discharge through phone interview (follow-up). The questionnaire was written in Traditional Chinese language, which was the first language of all patients. Informed consent was signed and obtained from every study participant and their legal guardians as required. Ethical approval was obtained from the ethics review board of the joint NTEC/CUHK Ethics Committee.

### SRS-30 questionnaire

All patients were required to fill out the first 23 questions (section 1), and question 24 to 30 (section 2) were for post-surgery patients. The 30 questions covered 5 domains: function/activity, pain, self-image/appearance, mental health, and satisfaction with management. Answers followed a 5-point Likert scale describing the degree of satisfaction with quality of life (between favourable and unfavourable opinions). Calculation of domain scores followed the suggestion scheme from SRS. A 5-point scale (5 = best outcome, 1 = worst outcome) is used for the calculation of domain scores.

### Statistical analysis

Gender-specific descriptive analyses and linear regression modelling were carried out. Surgery related parameters were tabulated by gender. Mean scores in different domains were calculated at 3 time points. Percentages of individual scores in the 5 domains at the 3 time points were tabulated. Pearson's correlations on scores collected at the 3 time points and "change after discharge (follow-up minus discharge)" were also carried out. Effects of potential risk factors (age, pre-op maximum Cobb angle, curve correction after surgery in degrees) on mean domain scores in male and female patients at the 3 time points were evaluated by linear regression models. All statistical analysis were performed using IBM SPSS version 20.0 (IBM Corp, Somers, NY). A two-sided p value ≤ 0.05 was considered statistically significant.

## Results

The mean age was 16.25 years at surgery, and 80.8% were female (Additional files 1). Follow-up (return) rate was 91.3%, of which 94.7% had been followed for more than one year. Male patients had larger maximum pre-op (p=0.03) and post-op (p=0.02) Cobb angles than female patients. There was a significant negative correlation between pre-op and discharge scores in function/activity (r=-0.47, p=0.05) in males. In females, correlations were found between pre-op and "change after discharge" in pain (r=-0.23, p=0.04), and satisfaction with management between pre-op and discharge (r=0.33, p<0.01) and pre-op and "change after discharge" (r=-0.47, p<0.01). No gender difference in all 5 domain scores at the 3 time points was found (Additional files 2). When including only the patients scoring all 5 scales, thereafter the percentages of patients scoring from 1 to 5, showed female patients reported significantly more pain (higher scores correspond to higher pain levels) before surgery (p<0.01) (Additional files 3). Moreover, male patients felt worse on their self-images before surgery (p=0.04).

Linear regression analysis showed that pre-op maximum Cobb angle was a significant predictor (r=-0.027, p=0.02) on satisfaction with management at follow-up in male patients (Additional files 4). Comparing the scores at "change after discharge" in male showed degree of curve correction after surgery was a significant predictor in self-image/appearance (r=-0.159, p<0.01) and satisfaction with management (r=-0.123, p<0.01). Pre-op maximum Cobb angle was found to be another significant predictor (r=0.052, p=0.02) on self-image/appearance in male. In female patients, degree of curve correction after surgery was a significant predictor (r=0.045, p=0.04) on function/activity at "change after discharge".

## Discussion

We found that there were gender differences on domain scores. Female patients demonstrated significant correlations on pain and satisfaction with management before and after surgery, and male patients on function/activity. Degree of curve correction after surgery and pre-op maximum Cobb angle were significant predictors of function/activity, self-image/appearance, and satisfaction with management in AIS patients.

No significant difference was observed when comparing individual domain scores between male and female patients, at all three time points. This reflects that male and female adolescents have similar feelings and self-awareness in all 5 domains. The effect is still the same after surgery.[3] For male patients, however, we found significant correlations between pre-op and after surgery (discharge or follow-up) in function levels, self-image, pain level and satisfaction with management in female patients. Medical professionals should pay attention to the difference in personal perceptions and feelings between boys and girls. The difference in the severity of scoliosis might limit the data generalizability of this study, however, males presenting Cobb angle 30 degrees or more showed a tendency towards curve progression irrespective of age.[9] Males always presented the symptom at relatively late stage at their first medical consultation, leading to the rapid curve progression and thus larger maximum pre-operative Cobb angle in males. In summary, we believe this is the first clinical study in Chinese community using the SRS-30 questionnaire. More resources and support for psychological management of patients-in-need should be allocated, and carefully managed at follow-up.

Pre-op maximum Cobb angle and corrections of Cobb angles after surgery were two main risk factors on self-image and satisfaction with management, particularly in male patients. Probably the reason is that male patients always had larger pre-op maximum Cobb angle than female patients, which made the degree of surgical correction difficult to match with female patients. Special care should be allocated to AIS patients, and try to arrange earlier surgical intervention, which prevents the ever-progressing spinal curvature making surgical treatment difficult at an advanced stage.

## Conclusions

Gender differences were found in which female patients demonstrated correlations with pain and satisfaction with management before and after surgery, and male patients with function/activity. Degree of curve correction after surgery and pre-op maximum Cobb angle were significant predictors of function/activity scores, self-image/appearance, and satisfaction with management in AIS patients. Allocation of resources for taking care of patients with problems of self-image and function should take these findings into account.

This is the extended abstract of IRSSD 2014 program book [10].

## Competing interest

The authors declare that they have no competing interests.

## Authors' contributions

Every author performed substantial contributions to conception and design, or acquisition of data, or analysis and interpretation of data.

BKWN, WWC, JCYC, TPL drafted the article or revised it critically for important intellectual content and obtained final approval of the version to be published.

## Supplementary Material

Additional files 1 Demographic characteristics of AIS patients by gender Click here for file

Additional files 2 Mean scores of individual domains and composite scores at Pre-op, Discharge, and Follow-up by gender Click here for file

Additional files 3 Percentages of patients scoring the 5 domains at Pre-op, Discharge, and Follow-up by gender Click here for file

Additional files 4 Linear regression models on the effect of potential risk factors on individual mean domain scores Click here for file

## Declarations

Publication charges for this collection were funded by IRSSD 2014 Sapporo.

This article has been published as part of *Scoliosis* Volume 10 Supplement 2, 2015: Research into Spinal Deformities 9: Short Papers from the IRSSD 2014 Meeting. The full contents of the supplement are available online at http://www.scoliosisjournal.com/supplements/10/S2.
